# Mapping gut parasitism patterns in a cohort of Egyptians

**DOI:** 10.1038/s41598-023-36320-z

**Published:** 2023-06-20

**Authors:** Eman S. El-Wakil, Rabab S. Zalat, Ayman A. El-Badry

**Affiliations:** 1 Department of Parasitology, Theodor Bilharz Research Institute, Kornaish El-Nile St., Giza, 12411 Egypt; 2 Department of Microbiology-Medical Parasitology Section, College of Medicine, Imam Abdulrahman Bin Faisal University, Dammam, Saudi Arabia

**Keywords:** Microbiology, Diseases

## Abstract

In developing countries, the prevalence of intestinal parasitic infection is still significant, particularly due to geographical and socioeconomic variables. The objective of this study was to map the distribution pattern of intestinal parasitic infection in a cohort of the Egyptian population, as well as to assess associated risk factors. A cross-sectional hospital-based study was conducted on 386 patients. A single fecal specimen was collected from the study individual and examined microscopically for the detection of parasitic infection. DNA was extracted from all samples and utilized to amplify *Entamoeba histolytica* complex species*, Cryptosporidium* species, *Giardia intestinalis* assemblages, and *Blastocystis* species using PCRs. Typing of *Cryptosporidium species* and* Giardia intestinalis* assemblages was performed using restriction enzymes *RasI* and *HaeIII* respectively. While *Blastocystis* spp. subtypes (ST) were identified through sequencing of PCR products and phylogenetic analysis. 59.6% (230/386) of the study patients were infected with one or more intestinal parasites, 87.4%; 201/230 of patients had mono-parasitic infections, and 12.6%; 29/230 had multiple-parasitic infections (*P* < 0.0001). The predominant protozoa were *Blastocystis*, followed by *Entamoeba histolytica* complex, and *Giardia intestinalis* both as mono-parasites and as part of multiple parasites. Molecular assays showed that *Blastocystis* ST3, *Entamoeba dispar*, *Giardia intestinalis* assemblage B, and *Cryptosporidium hominis* were the most prevalent species. Intestinal parasitic infection was significantly associated with age, gender, residence, and water source. Multi-parasitism showed that residency in a rural area was a risk factor (OR 4.49; 95% CI 1.51–13.37; *P* = 0.007). Egyptians residing in rural areas have a high prevalence of intestinal multi-parasitism. Therefore, to lessen the prevalence and effects of these infections in this group, effective and sustainable control methods, providing health education focusing on good personal hygiene habits, and providing a safe drinking water supply should be implemented.

## Introduction

Intestinal parasites (IPs) are widespread throughout the world and have a significant negative influence on morbidity and mortality, particularly in developing countries^1,2^. IPs have been linked to stunting, physical weakness, and poor academic performance in children. By interacting with absorptive surfaces, physically obstructing the intestinal lumen, producing proteolytic chemicals, and consuming nutrients destined for the body, IPs reduce nutrient intake in the body. Due to their heightened susceptibility to nutritional inadequacies, children are more vulnerable to the effects of intestinal parasitic infections^3,4^. Furthermore, IPs-related disorders display a heavy burden. Over two billion people are affected globally by IPs, of which 300 million have severe morbidity and approximately 4.5 billion people are at risk. The World Health Organization reported that there were 200 million instances of *Giardia lamblia*, 800 million cases of *Ascaris lumbricoides*, 700 million cases of hookworm, 500 million cases of *Trichuris trichuria*, and 500 million cases of *Entamoeba histolytica*/ *dispar* worldwide. According to earlier estimates, IPs result in an annual loss of 39 million disability-adjusted life years, resulting in serious health and financial problems. However, the biggest problem of IPIs is that 90% of infected people are asymptomtic^5,6^.

The main intestinal protozoans that infect humans are *Entamoeba histolytica*, *Balantidium coli*, *Giardia lamblia*, *Isospora belli*, and *Cryptosporidium* species^7^. Other species, like *Blastocystis sp*. and *Dientamoeba fragilis*, are disputed pathogens, their presence is not associated with clinical significance and their role in people who have digestive symptoms is currently debatable. However, few clinics and laboratories routinely test for *Dientamoeba fragilis* and *Blastocystis* sp.^8^. Similarly, many other non-pathogenic intestinal protozoa can be found in humans that are not associated with diseases, including *Entamoeba coli*, *Entamoeba dispar*, *Entamoeba hartmanni*, *Entamoeba polecki*, *Endolimax nana*, *Iodamoeba bütschlii,* and *Chilomastix mesnili*^9^. Intestinal helminths including *Ascaris lumbricoides (A. lumbricoides)*, *Trichuris trichiura (T. trichiura)*, and hookworm species (*Necator americanus* and *Ancylostoma duodenale*) are grouped under soil-transmitted helminths, which are highly prevalent in developing countries^10^. In Egypt, intestinal schistosomiasis (*S. mansoni*) is prevalent in the Nile Delta while urogenital schistosomiasis (*S. haematobium*) in the Nile valley south of Cairo, down to Aswan and beyond. Epidemiological studies have shown that in endemic areas, multiparasitism is more frequent than single infections^11–13^. Geographical, behavioral, biological, and socioeconomic factors all influence how prevalent these illnesses are. IPs are directly related to the hot and humid climate of the tropics, the lack of access to clean water, the unsanitary environmental conditions, overcrowding, and low family income. These circumstances facilitate intestinal parasite growth, transmission, and exposure^14^.

The majority of research on co-infections with several parasites, which occurs when infectious agents coexist in the same host and can have a prevalence of up to 80% in some populations, has focused on humans^15^. These co-infections may result from a variety of environmental factors, such as contagious settings and transmission paths, and may be aided or hindered by direct or indirect interactions between different parasite species^16^. The interaction between different parasite populations (within and between hosts) and host populations, rather than associations play a significant role^17^. Though the specifics of these interactions within the host are unknown, some are sought to be synergistic where the presence of one parasite may encourage the occurrence of future parasitic infections, while others are thought to be antagonistic where parasites compete for the same ecological niche within the host^18^.

Studies carried out in several governorates over the past few years to determine the prevalence of IPs and the risk factors associated with them among the Egyptian population have found that IPs continue to be a problem for public health. Therefore, for adequate planning and execution of efficient preventative and control strategies, frequent assessment of IPs prevalence and identification of the related risk factors is required. Therefore, the current study’s objective was to investigate the prevalence of multiparasitism and intestinal parasites. Additionally, to study the relationships between multiple parasite infections and their risk factors in comparison to single parasite infections or complete lack of infection in the Egyptian population.

## Results

The diagnostic yield of microscopy and molecular assays to document the presence of intestinal parasites in patients’ fecal samples were shown in Table 1 and Supplementary figure.

**Table 1:** Diagnostic yield of intestinal parasites using coproscopy.

	Frequency		
	Asymptomatic	Symptomatic	Total
Mono-parasite			
*Blastocystis* species	48 (43.6%)	62 (56.4%)	110 (47.8%)
*Entamoeba histolytica* complex	30 (50%)	30 (50%)	60 (26%)
*Giardia* intestinalis	1 (4.3%)	23 (95.7%)	24 (10.4%)
*Cryptosporidium* species	2 (66.7%)	1 (33.3%)	3 (1.2%)
*Entrobius vermicularis*	1 (33.3%)	2 (66.7%)	3 (1.2%)
*Hymenolepis nana*	0 (0.0%)	1 (100%)	1 (0.4%)
Total	82 (40.8%)	119 (59.2%)	201 (87.4%)
Multiple parasites			
Two parasites			
*Blastocystis* species + *Entamoeba* histolytica complex	0 (0.0%)	10 (100%)	10 (4%)
*Blastocystis* species + *Entamoeba* coli	1 (11.1%)	8 (88.9%)	9 (3.6%)
*Blastocystis* species + *Giardia* intestinalis	0 (0.0%)	3 (100%)	3 (1.2%)
*Entamoeba histolytica* complex + *Giardia* intestinalis	0 (0.0%)	3 (100%)	3 (1.2%)
*Entamoeba histolytica* complex + *Entamoeba coli*	1 (50%)	1 (50%)	2 (0.8%)
Three parasites			
*Blastocystis* species + *Entamoeba histolytica* complex + Entamoeba coli	0 (0.0%)	1 (100%)	1 (0.4%)
*Blastocystis* species + *Giardia intestinalis* + *Hymenolepis nana*	0 (0.0%)	1 (100%)	1 (0.4%)
Total	2 (6.9%)	27 (93.1%)	29 (12.6%)

*P* value = 0.033.

Intestinal parasites were microscopically detected in the stool specimens of 230 patients among the examined 386 Egyptian patients with a prevalence rate of 59.6%. *Blastocystis* species was the predominant intestinal parasite, both as a mono-parasites (110/230 [47.8%]) or part of a multi-parasites (24/230 [10.4%]). Single intestinal parasites were detected in 87.4% (201/230) of the microscopically examined stool specimens. There was a clear predominance of intestinal protozoa (225/230 [97.8%]) compared to intestinal helminths (5/230 [2.2%]). *Blastocystis* was the predominant protozoa (47.8%), followed by *Entamoeba histolytica complex* (26%), and *Giardia intestinalis* (10.4%). Multiparasitism was detected in 12.6% (29/230) of the patients, 27 (11.8%) of them had two parasites and 2 (0.8%) of them had three parasites.

The prevalence of parasites was confirmed by PCR, *Blastocystis* copro-DNA was detected in 139 (60.4%), of which 134 (58.2%) were positive by microscopy, no samples were positive by microscopy, and negative by PCR. The diagnostic performance of *Blastocystis* showed that the sensitivity of microscopy was 96.40% compared to PCR results.

On the other hand, 27(11.7%) and 68(29.6%) of the examined specimens were molecularly positive for *Giardia intestinalis* and *Entamoeba histolytica complex*, respectively while, 31 (11.9) and 76 (33%) were positive microscopically (Table 2). The diagnostic performance of *Giardia intestinalis* and *Entamoeba histolytica complex* microscopy in diagnosis displayed a sensitivity of 100% in comparison with PCR which showed a sensitivity of 87.10%, and 89.47% respectively.

**Table 2:** Diagnostic yields of microscopy and PCR assay for detection of *Giardia intestinalis* among study cohort.

	Microscopy		
	Positive	Negative	Total
nPCR-RFLP			
Positive			
Assemblage A	6	0	6
Assemblage B	21	0	21
Total	27	0	27
Negative	4	355	359
Total	31	355	386 (100%)

*Cryptosporidium* species were in three stool specimens by both microscopy and nested PCR with the same sensitivity, all of which were *Cryptosporidium hominis. Giardia intestinalis* assemblages A and B were detected, assemblage B was the predominant assemblage (21/27 [77.8%]).

*Entamoeba histolytica* complex species were molecularly identified using multiplex PCR in 68 patient samples. *Entamoeba histolytica* (20.6%; 14/68) was detected in one-fifth of the molecularly positive samples (Table 3). Regarding the nonpathogenic species, *Entamoeba dispar* was the most common species (44.1%; 30/68), followed by *Entamoeba moshkovskii* (35.3%; 24/68).

**Table 3:** Diagnostic yield of microscopy and PCR assay for detection of *Entamoeba histolytica* complex species among study individuals.

	Microscopy		
	Positive	Negative	Total
nPCR-RFLP			
Positive			
*Entamoeba histolytica*	14	0	14
*Entamoeba dispar*	30	0	30
*Entamoeba moshkovskii*	24	0	24
Total	68	0	68
Negative	8	310	318
Total	76 (19.7%)	310 (80.3%)	386 (100%)

Only ten PCR products positive for *Blastocystis* showed high-quality sequences, among them three STs were detected (ST1, 2, and 3). The three detected STs displayed more or less equal distribution with ST3 being the most prevalent ST among the sequenced samples (4/10; 40%) followed by ST1 and ST2 (30% (3/10) each).

**Figure 1:**
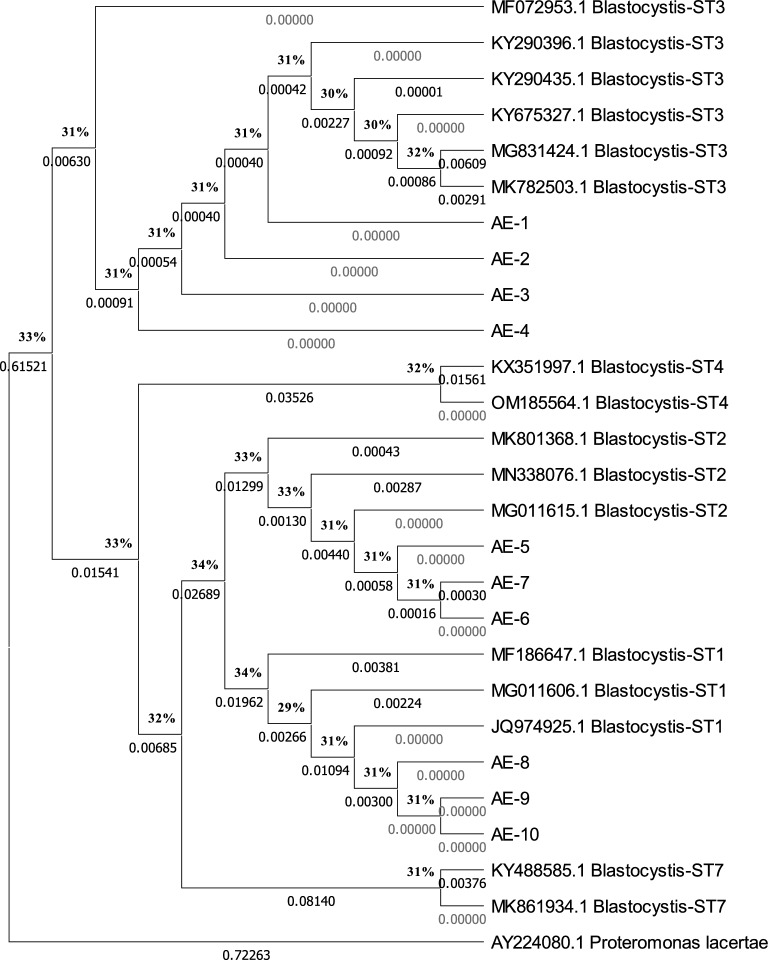
Dendrogram representing the neighbor-joining phylogenetic tree of the three *Blastocystis* subtypes (start with the abbreviation AE followed by the sample number) from our study patients compared to reference subtypes from the GenBank (the accession number is written before their names and subtypes). The bootstrap analysis is based on 1000 replicates.

All obtained sequences were deposited in the GenBank database with accession numbers (OR015950–OR015959) (Fig. 1).

The age of the study individuals ranged from 3 months to 75 years with a mean and standard deviation of 29.17 ± 18.83. Most of the infected participants were male (59.1%), rural residents (61.7%), and consuming tap water (70%). There were statistically significant differences between infected and non-infected participants regarding age, gender, residence, and water source (Tables 4, 5, 6). The most-reported GIT symptoms among infected participants were abdominal pain (51.3%), and diarrhea (37.8%), with highly significant differences between infected and non-infected participants (Tables 4, 5, 6).

**Table 4:** Univariate analysis of risk factors of intestinal parasitic infection among study individuals.

Variables	Infected N (%)	Non-infected N (%)	Total N (%)	OR (95% CI(	*P* value
Age groups					
Infants	2 (0.87%)	2 (1.3%)	4 (1.1%)	.760 (.074–7.821)	.211
Preschool child	20 (8.7%)	7 (4.5%)	27 (7.0%)	.390 (.116–1.313)	
School child	55 (23.9%)	27 (17.3%)	82 (21.2%)	.470 (.180–1.230)	
Adolescent	44 (19.1%)	27 (17.3%)	71 (18.4%)	.522 (.203–1.340)	
Young adult	46 (20.03%)	34 (21.8%)	80 (20.7%)	.434 (.167–1.123)	
Middle-aged adult	50 (21.7%)	47 (30.1%)	97 (25.1%)	.752 (.301–1.878)	
Old adult	13 (5.7%)	12 (7.7%)	25 (6.5%)	.654 (.402–1.756)	
Gender					
Male	136 (59.1%)	73 (46.8%)	209 (54.1%)	.566 (.359–.892)	.014
Female	94 (40.9%)	83 (53.2%)	177 (45.9%)	1.44 (.211–1.877)	
Residence					
Urban	88 (38.3%)	86 (55.1%)	174 (45%)	1.519 (.939–2.460)	.089
Rural	142 (61.7%)	70 (44.9%)	212 (55%)	1.62 (1.55–1.68)	
Water source					
Unfiltered tap water	161 (70%)	89 (49.4%)	250 (64.8%)	1.430 (.089–2.881)	.648
Filtered water	52 (22.6%)	57 (44.2%)	109 (28.2%)	1.910 (.119–3.672)	
Bottled water	17 (7.4%)	10 (6.4%)	27 (7%)	1.662 (.114–3.880)	
Animal contact					
Yes	101 (43.9%)	79 (50.6%)	180 (46.6%)	1.99 (1.98–2.00)	.115
No	129 (56.9%)	77 (49.4%)	206 (53.4%)	1.98 (1.97–2.00)	
Clinical symptoms					
Vomiting					
Yes	2 (0.9%)	5 (3.2%)	7 (1.8%)	7.945 (1.117–16.528)	.038
No	228 (99.1%)	151 (96.8%)	379 (98.2%)	3.44 (1.013–6.235)	
Fever					
Yes	2 (0.9%)	2 (1.3%)	4 (1%)	2.202 (.187–2.905)	.530
No	228 (99.1%)	154 (98.7%)	382 (99%)	2.011 (.197–2.665)	
Abdominal pain					
Yes	118 (51.3%)	46 (29.5%)	164 (42.5%)	.425 (.266–.680)	.000*
No	112 (48.7%)	110 (70.5%)	222 (57.5%)	.077 (.021–.741)	
Diarrhea					
Yes	87 (37.8%)	26 (16.7%)	113 (29.3%)	.325 (.187–.566)	.000*
No	143 (62.2%)	130 (83.3%)	273 (70.7%)	.124 (.051–.433)	

Data presented as n and percentage with (*) *P* value ˂ 0.05 is significant.

**Table 5:** Regression analysis of the variables.

ANOVA					
Model	Sum of Squares	df	Mean Square	F	Sig
1Regression	13.743	10	1.374	6.506	.000
Residual	79.210	375	.211		
Total	92.953	385			

From the above table, we conclude that there is a significant effect of risk factors on intestinal parasitic infection where (sig = 0.00) and (F = 6.506).

**Table 6:** Regression analysis of the variables.

Coefficients					
Model	Unstandardized coefficients		Standardized coefficients	t	Sig
	B	Std. Error	Beta		
1 (Constant)	1.698	1.453		1.168	.243
Age	.030	.016	.090	1.839	.067
Sex	.116	.048	.118	2.442	.015
Residence	− .094	.051	− .095	− 1.821	.069
Fever	− .217	.234	− .045	− .926	.355
Abdominal pain	.173	.049	.174	3.545	.000
Diarrhea	.212	.053	.196	3.984	.000
Vomiting	− .429	.188	− .117	− 2.275	.023
Unfiltered Tap water	.007	.259	.007	.026	.979
Filtered water	− .059	.260	− .054	− .225	.822
Bottled water	.147	.236	.082	.622	.535

From the above table, we conclude that there are none of the patients’ characteristics was a statistically significant risk factor for IPs infections.

The association between infection with one parasite or more than one parasite (multiparasitism) and the individuals’ sociodemographic and clinical data was studied (Table 7). Participants with poly-parasites had higher odds of reporting sociodemographic data such as residence in a rural area and animal contact compared to participants with mono-parasitic infection (*P* < 0.05). There was a trend for a higher likelihood of abdominal pain and diarrhea among individuals with poly-parasitic infection than mono-parasitic infection (*P* < 0.05). No association was found between polyparasitic infection and (age, gender, water source, vomiting, and fever) (*P* > 0.05).

**Table 7:** Univariate analysis of mono and poly parasitic infection risk factors.

Variables	Mono-parasite N (%)	Poly-parasites N (%)	OR (95% CI(	*P* value
Age	28.92 ± 18.67	30.87 ± 19.77	1.95 (− 5.41 to 9.31)	0.602
Age groups				
Infants	2 (1.0%)	0 (0.0%)	NA	0.890
Preschool child	18 (9.0%)	2 (6.9%)		
School child	48 (23.8%)	7 (24.1%)		
Adolescent	39 (19.4%)	5 (17.2%)		
Young adult	42 (20.9%)	4 (13.8%)		
Middle-aged adult	40 (19.9%)	10 (34.5%)		
Old adult	12 (6.0%)	1 (3.5%)		
Gender				
Male	118 (58.7%)	18 (62.1%)	0.868 (0.39–1.94)	0.731
Female	83 (41.3%)	11 (37.9%)		
Residence				
Urban	84 (41.8%)	4 (13.8%)	4.49 (1.51–13.37)	0.007*
Rural	117 (58.2%)	25 (86.2%)		
Water source				
Unfiltered tap water	135 (67.1%)	26 (89.7%)	NA	0.069
Filtered water	49 (24.4%)	3 (10.3%)		
Bottled water	17 (8.5%)	0 (0%)		
Animal contact				
Yes	80 (39.8%)	23 (79.3%)	0.173 (0.067–0.442)	0.0003*
No	121 (60.2%)	6 (20.7%)		
Clinical symptoms				
Vomiting				
Yes	1 (0.5%)	1 (3.4%)	0.140 (0.008–2.301)	0.169
No	200 (99.5%)	28 (96.6%)		
Fever				
Yes	1 (0.5%)	1 (3.4%)	0.140 (0.008–2.301)	0.169
No	200 (99.5%)	28 (96.6%)		
Abdominal pain				
Yes	97 (48.3%)	21 (72.4%)	0.355 (0.150–0.839)	0.018*
No	104 (51.7%)	8 (27.6%)		
Diarrhea				
Yes	63 (31.3%)	24 (82.8%)	0.095 (0.035–0.261)	< 0.0001*
No	138 (68.7%)	5 (17.2%)		
Total N (%)	201 (87.4%)	29 (12.6%)		

## Discussion

This study determined the prevalence of intestinal parasitic infection among a cohort of Egyptians, in addition to investigating mono- and poly-parasitic patterns with their associated risk factors. Epidemiological studies are essential for determining the prevalence of intestinal parasitic infections, identifying risk factors, and creating effective prevention and control strategies, all of which continue to be a major health concern in developing countries such as Egypt^19^.

There was a relatively high prevalence of intestinal parasites (59.6%) in the studied cohort of Egyptians which was similar to a study by Omar and Abdelal^20^, who reported a 56% prevalence of intestinal parasitic infection among Egyptian patients. Ahmed and Abu-Sheishaa^19^ reported that IPs represented an overall prevalence of 32.9% among school children. This variation in findings may be due to differences in sociodemographic and environmental factors with different exposures to risk factors.

Of the infected individuals, 201 (87.4%) had mono-parasite, 29 (12.6%) had multiple-parasites with double infection (27, 11.8%), and triple infection (2, 0.8%), which can be explained by sharing the same social-ecological contexts that encourage the occurrence and transmission of these parasitic diseases. In terms of the detected intestinal parasites, *Blastocystis* was the predominant parasite, for both a mono-infection (110, 48.7%) and coinfection (24, 10.4%), followed by *Entamoeba histolytica* complex (26%), and *Giardia intestinalis* (10.4%). This finding is consistent with a study conducted by Diarthini in Karangasem (Bali, Indonesia), where *Blastocystis spp*. was shown to be the most common parasite among primary school students^21^. In poly-parasitic infections, *Blastocystis spp*. was the most common protozoa. This finding was consistent with a study by Diarthini et al.^21^ that identified *Blastocystis spp*. to be frequently present in poly-parasitic infections with *Giardia intestinalis*,* Entamoeba histolytica* complex, and *Entamoeba coli*. In contrast, previous studies conducted in Egypt reported *Giardia intestinalis* as the most prevalent parasitic infection^22,23^.

In this study, molecular methods for identifying human infections and protozoa were standardized and evaluated. The minimum amount of DNA per µL or per PCR reaction was used to determine the sensitivity of all standardized molecular techniques (PCR, nested or semi-nested PCR, PCR–RFLP). Where sensitivity is the smallest number of living forms that can be detected by molecular assays. Standardized tests made it possible to identify species of the same genus that were not distinguishable by microscopic examination, as in the case of the differentiation between *E. histolytica*, *E. dispar*, and *E. moshkovskii*, as well as to identify genotypes (*Blastocystis spp*.) or assemblages (*G. intestinalis*) that were crucial for using molecular epidemiology or transition studies.

Depending on the method chosen for parasite identification, prevalence data can readily be changed, either positively or negatively. Laboratory diagnosis of *Blastocystis* can be fairly difficult. Despite being the preferred method for diagnosing *Blastocystis* all around the world, direct microscopic examinations have limited applications in clinical microbiology labs and epidemiological studies. Polymorphic creatures and inanimate objects at wet mounts may be mistaken for other living things. When compared to PCR, our investigation showed that direct wet mount had a less sensitivity (96.4%). The three detected *Blastocystis* STs displayed more or less equal distribution with ST3 being the most prevalent ST among the sequenced samples. This result agrees with Ahmed et al.^20^ who reported three *Blastocystis* STs (ST1, ST2, and ST3), with ST3 (45.5%) representing the most common subtype. DNA sequencing and phylogenetic analysis of Egyptian *Blastocystis* isolates identified three different subtypes. Further studies are required to determine the distribution of STs in the general population. Nonetheless, our study is a contribution to the understanding of the molecular epidemiology, transmission patterns, and genetic diversity of *Blastocystis*.

With the use of Lugol, the cysts of *Entamoeba spp*. can be identified using light microscopy based on their morphological traits^24^. Cysts of the pathogenic amoeba *E. histolytica* resemble the non-pathogenic amoebae *E. moshkovskii* and *E. dispar* in terms of morphology. Other molecular epidemiology studies have identified *E. moshkovskii* as the causative agent of gastrointestinal symptoms using multiplex PCR and the detection of ribosomal SSU identical to 16S^25^. Our investigation showed that PCR had (89.47%) less sensitivity. In this study, concerning *Entamoeba histolytica* complex, our findings demonstrated that most samples were attributed to *Entamoeba dispar* and *Entamoeba moshkovskii*. This result is in line with previous studies carried out in Egypt by El-Badry et al.^26^, and in Iran, by Fallah et al.^27^ who reported *Entamoeba dispar* as the most prevalent *Entamoeba* species. In contrast, a study conducted in the United Arab Emirates found *Entamoeba histolytica* to be more prevalent^28^. In the present study, there were four cases of co-infections, with the three different *Entamoebae* species.

In this study, *G. intestinalis* was identified from fecal samples using morphological and molecular detection methods. *Giardia* diagnosis in medical labs is based on wet mount preparation of patient feces and ocular detection by light microscopy. These procedures must be carried out by qualified microscopists and medical laboratory technicians. On the other hand, these methods may not be sensitive enough to find small quantities of expelled *Giardia* cysts in fecal samples and are unable to distinguish between the genetic assemblages of *G. intestinalis* isolates. To find *G. intestinalis* cysts in feces, molecular detection methods based on PCR have been developed. Our investigation showed that PCR had (87.1%) less sensitivity. *G. intestinalis* cysts can be genotyped using these molecular techniques. Genotyping analysis for *Giardia intestinalis* showed assemblage B predominance (77.8%), this result is similar to previous studies in Egypt that reported the predominance of assemblage B^29–31^. Genotyping for *Cryptosporidium* revealed only* cryptosporidium hominin*.

Regarding age, gender, residence, and water source, there was a statistically significant difference between infected and non-infected individuals. These study findings are similar to previous studies which revealed significant differences regarding age^32–34^, gender differences^33,35^; which can be explained by considerable outdoor activity and increased exposure to infectious diseases, and in rural areas^36,37^, this can be explained by their risky behavior, such as interaction with domestic animals, poor hygiene, defecating in an open field of agriculture near water sources, all of which increase the level of parasite contamination in the soil^38^. Also, there were highly significant differences between the infected and non-infected individuals regarding the GIT symptoms (abdominal pain and diarrhea) (*P* < 0.0001). These findings agree with the findings of similar studies^39^. As expected, we observed a significantly higher multiparasitism infection rate among participants with gastrointestinal symptoms compared to the asymptomatic group.

In the current study, polyparasitism was found to be more common among males than females, but this difference was not statistically significant. In contrast, a study from Nigeria revealed that females are more vulnerable to polyparasitism than males^35^. The variation seems to support the idea that male outdoor activities may increase relative interaction with the parasitic infectious stages. Our findings showed the risk for polyparasitism significantly increased with residency in rural communities. This finding is in agreement with reports from other parts of Africa, especially the rural communities^40,41^. Moreover, animal contact showed a highly significant association with polyparasitism among our study individuals. This could be attributed to the zoonotic nature of the parasitic infection which is still unresolved in literature. Also, there was a significant association between polyparasitism and both abdominal pain (*P* = 0.018) and diarrhea (*P* < 0.0001). These findings agreed with Suwarna Pawar et al.^42^ who reported that multiple parasitic infestations in low socioeconomic populations can be a cause of diarrhea.

In the current study, a major strength was the inclusion of a wide spectrum of age groups, as this facilitates the comparison of infection prevalence and their association with age. On a broader scale, the identification of individuals with polyparasitism using molecular tools, such as PCR, is important as it can provide a measure of disease burden and also serve as a guide for the development of specific prevention and control interventions against multiple parasites. Our results contribute toward mapping the molecular epidemiology, transmission patterns, and genetic diversity of parasitic infections at a regional and global scale.

Our study was limited by being a single center institutional study including patients attending TBRI which could be the reason that there were relatively few worm infections and many protozoans, and very few *Cryptosporidium*. Another limitation is the lack of data concerning certain risk factors, including socioeconomic status, educational level of patients, and family history of intestinal symptoms that would help in the detection of prevalence-associated risk factors.

## Conclusion

Based on our study findings, the studied cohort of Egyptians had a relatively high rate of intestinal parasitism. The most prevalent parasites were *Blastocystis,* followed by *Entamoeba histolytica complex,* and* Giardia intestinalis.* Age, gender, residing in a rural area, and water source were all potential risk factors for intestinal parasitism. Additionally, there was a significantly substantial correlation between GIT symptoms and parasite infection. One-eighth of our research population, particularly in rural Egypt, had multiple intestinal parasitic infections. Understanding the distribution pattern of intestinal parasites would help thus in the strategic planning of prevention and control measures.

## Methods

### Study design and population

A cross-sectional study was conducted from April 2020 to May 2021. A single stool specimen was collected from 386 Egyptian, aged from 3 months to 75 years, attending Theodor Bilharz Research Institute (TBRI) clinics, having gastrointestinal symptoms, or as part of a routine check-up. A questionnaire was used to collect relevant sociodemographic and clinical data. Participants taking antiparasitic treatment within the last 2 months were excluded from the study.

### Sample size determination and sampling technique

Using the general formula,* n* = *z*^2^*p* (1 − *p*)/d^2^, the sample size was calculated with a 95% confidence level, a 5% margin of error, and a 21% response distribution. The calculated sample size was 285 samples^43^. The used sample size was 314 people accounting for a 10% non-response rate.

### Copro-parasitological examination

All collected stool specimens were examined microscopically using both saline solution and Lugol temporary stain, before and after using the formalin-ethyl acetate concentration method^44^ to detect fecal parasitic stages. Feces were smeared and stained by a modified acid-fast (AF) stain for the detection of *Cryptosporidium* and other coccidian oocysts^45^.

### Molecular assays

#### Extraction of genomic DNA

After the initial thermal shock (10 cycles of freezing in liquid nitrogen and thawing at 95 °C) of fecal specimens, DNA extraction was performed using the DNA Stool Mini Kit (QIAGEN, Hilden, Germany), according to the instructions of the kit.

#### PCR assays

Extracted copro-DNA were amplified using three PCRs, (1) multiplex PCR (m-PCR) to detect the three *Entamoeba histolytica* complex species; *Entamoeba histolytica*,* Entamoeba dispar* and *Entamoeba histolytica Entamoeba moshkovskii* (2) two nested PCR to detect *Cryptosporidium species* and *Giardia intestinalis*, and (3) conventional PCR to detect *Blastocystis species*. The used primer sequences and PCR reaction conditions of the used PCRs were listed in Table 8. The PCR products were visualized on 1.5% agarose gel and stained with ethidium bromide under a UV light system.

**Table 8:** Primers, targets, reaction conditions, and restriction enzymes.

Primer name	Sequence (5′–3′)	Gene	Reaction Conditions	References
EntaF	ATG CAC GAG AGC GAA AGC AT	(SSU rRNA) *Entamoeba histolytica* complex	35 cycles of 94 °C for 60 s, 58 °C for 60 s, and 72 °C for 80 s	^26^
EhR	GAT CTA GAA ACA ATG CTT CTC T			
EdR	CAC CAC TTA CTA TCC CTA CC			
EmR	TGA CCG GAG CCA GAG ACA T			
BcowpF	ACC GCT TCT CAA CAA CCA TCT TGT CCT C	*(COWP) Cryptosporidium*	35 cycles of 94 °C for 60 s, 63 °C for 60 s, and 72 °C for 60 s	^46,47^
BcowpR	CGC ACC TGT TCC CAC TCA ATG TAA ACC C			
Cry-15	GTA GAT AAT GGA AGA GAT TGT G		35 cycles of 94 °C for 60 s, 54 °C for 30 s, and 72 °C for 60 s	
Cry-9	GGA CTG AAA TAC AGG CAT TAT CTT G			
BG-newF1	AAGCCCGACGACCTCACCCGCAGTGC	*(b-giardin) Giardia intestinalis*	35 cycles of 94 °C for 30 s, 65 °C for 30 s, and 72 °C for 60 s	^48^
BG-newR1	GAGGCCGCCCTGGATCTTCGAGACGAC			
BG-newF2	GAACGAACGAGATCGAGGTCCG		35 cycles of 94 °C for 30 s, 52 °C for 30 s, and 72 °C for 60 s	
BG-newR2	CTCGACGAGCTTCGTGTT			
RD5	ATCTGGTTGATCCTGCCAGT	(SSU rRNA) *Blastocystis*	35 cycles of 94 °C for 60 s, 59 °C for 60 s, and 72 °C for 60 s	^49^
BhRDr	GAGCTTTTTAACTGCAACAACG			

Nested PCR-Restriction Fragment Length Polymorphism (RFLP).

*Cryptosporidium* genotypes and *Giardia intestinalis* assemblages were detected by the banding patterns in the restriction analysis of nested PCR products of *Cryptosporidium species* and *Giardia intestinalis* with restriction enzymes *RasI* and *HaeIII*, respectively. Digestion products were fractionated on 3% Metaphor agarose after ethidium bromide staining.

#### Sequencing and phylogenetic studies of *Blastocystis* spp

Thermo Scientific Gene JET PCR Purification Kit was used to purify the positive PCR products for *Blastocystis*, and sequencing was done using the primer pair RD5 and BhRDr, and Big-Dye® Terminator v3.1. Ready Reaction Cycle Sequencing Kit (Applied Biosystems, Foster City, CA, USA) was also used following the manufacturer’s instructions for the ABI Prism 310 genetic analysis. Sequences from *Blastocystis* isolates were corresponded with reference sequences in the GenBank database using online BLAST software at the National Center for Biotechnology Information (NCBI) (http://www.ncbi.nlm.nih.gov/BLAST). All sequences were aligned using the BioEdit software’s ClustalW tool^50^. The phylogenetic tree for the sequences was made using the neighbor-joining approach^51^ with the Molecular and Evolution Genetic Analysis X (MEGA10) program^52^. Bootstrapping was used to assess the trustworthiness of the phylogenetic tree (1000 replicates). The Maximum-Likelihood approach with the Tamura-3 parameter substitution model was used with MEGA10 to calculate the evolutionary distances.

### Statistical analysis

For descriptive data, the data are displayed as numbers and percentages. For categorical data, the chi-square and Fisher's exact tests were utilized to evaluate differences. The odds ratio and 95% confidence interval for each factor that affected the prevalence of intestinal parasite infection in the population under study were calculated using univariate analysis. The cutoff for significance was *P* < 0.05. The statistical program for social science (SPSS) Version 28 for Windows was used for all statistical analyses (SPSS Inc., Chicago, IL, USA).

### Ethical considerations

This study was performed after approval of the Research Ethical Committee of Theodor Bilharz Research Institute (TBRI-REC), with the serial number of protocol: PT (499). The TBRI-REC is registered at the Office for Human Research Protections, US Department of Health and Human Services, and operates under Federal Wide Assurance No. FWA00010609. All the approved research work complies with the World Medical Association Codes of Ethics (Declaration of Helsinki) for experiments with humans. Participant informed consent was obtained from all participants and/or their legal guardians.

## Supplementary Information

Supplementary Information.

## Data Availability

All data generated or analyzed during this study are included in this published article.
