# Supplementary material for: Mapping gut parasitism patterns in a cohort of Egyptians
Source: Sci Rep. 2023 Jun 20;13:9961. doi: 10.1038/s41598-023-36320-z (PMC10282082; doi:10.1038/s41598-023-36320-z)
Supplement: Supplementary file 1 — Supplementary Information. [file 41598_2023_36320_MOESM1_ESM.docx]

**Supplementary materials**

| 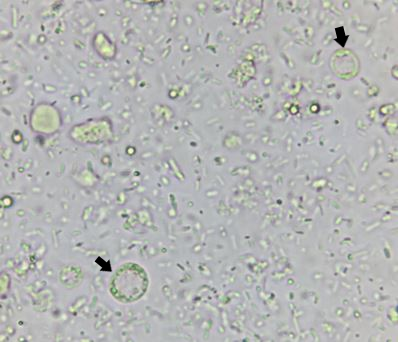  10 μl  **A** | 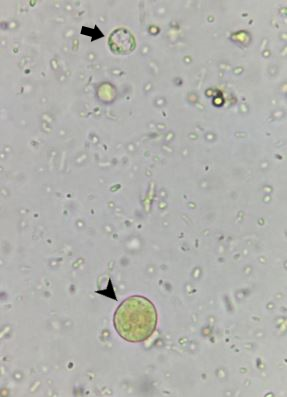  10 μl  **B** |
| --- | --- |
| 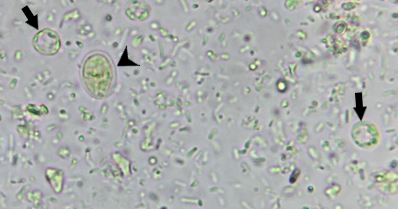  10 μl  **C** | 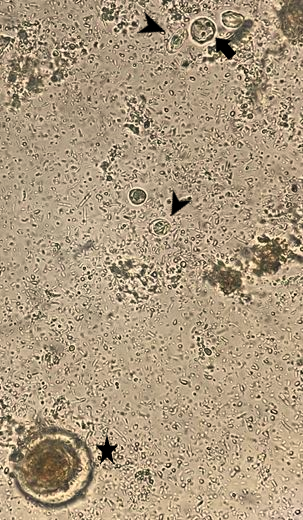  10 μl  **F**  **D** |
| 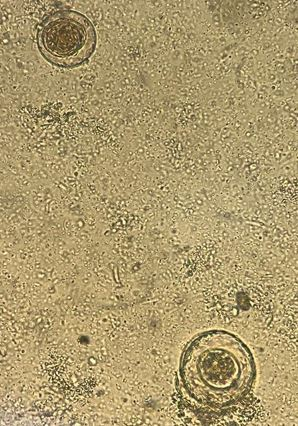  10 μl  **E** | 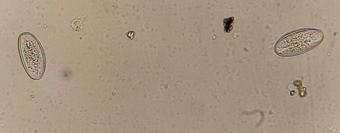  10 μl |

**Supplementary figure.** Different parasitic stages (A) *Blastocystis* species cyst (arrows); (B) *Entamoeba* *histolytica* complex cyst (arrowhead) mixed with *Blastocystis* species cyst (arrow); (C) *Giardia intestinalis* cyst (arrowhead) mixed with *Blastocystis* species cyst (arrows); (D) *Hymenolepis* *nana* egg (star) mixed with *Blastocystis* species cyst (arrow) and *Giardia intestinalis* cyst (arrowhead). (E) *Hymenolepis nana* eggs. (F) *Entrobius vermicularis* eggs. Photos were taken with×40.
